# Diabetes Therapeutics of Prebiotic Soluble Dietary Fibre and Antioxidant Anthocyanin Supplement in Patients with Type 2 Diabetes: Randomised Placebo-Controlled Clinical Trial

**DOI:** 10.3390/nu17071098

**Published:** 2025-03-21

**Authors:** Chompoonut Teparak, Juntanee Uriyapongson, Jatuporn Phoemsapthawee, Orathai Tunkamnerdthai, Ploypailin Aneknan, Terdthai Tong-un, Charnchai Panthongviriyakul, Naruemon Leelayuwat, Ahmad Alkhatib

**Affiliations:** 1 Exercise and Sport Sciences Program, Graduate School, Khon Kaen University, Khon Kaen 40002, Thailand; chompoonut.tep@kkumail.com; 2 Exercise and Sport Sciences Development and Research Group, Khon Kaen University, Khon Kaen 40002, Thailand; ploypailin.anek@gmail.com (P.A.); terdthai@kku.ac.th (T.T.-u.); 3 Department of Food Technology, Faculty of Technology, Khon Kaen University, Khon Kaen 40002, Thailand; juntanee@kku.ac.th; 4 Department of Sports Science, Faculty of Sports and Health Science, Kasetsart University, Nakhon Pathom 73140, Thailand; jatuporn.w@ku.th; 5 Department of Physiology, Faculty of Medicine, Khon Kaen University, Khon Kaen 40002, Thailand; torata@kku.ac.th; 6 Department of Pediatrics, Faculty of Medicine, Khon Kaen University, Khon Kaen 40002, Thailand; chapan@kku.ac.th; 7 College of Life Sciences, Birmingham City University, Birmingham B15 3TN, UK

**Keywords:** anthocyanin, rice bran, inulin, hyperglycaemia, dyslipidaemias, six-minute walk test

## Abstract

Background: Antioxidants and prebiotics are popular functional foods known for their distinct physiological ameliorating benefits on type 2 diabetes mellitus (T2DM). Whether and how a combined antioxidant-prebiotic supplement affects primary and secondary T2DM outcomes is not known. Objectives: We investigated the therapeutic effects of an antioxidant (anthocyanin from riceberry rice) combined with prebiotics (dietary fibre from rice bran and Jerusalem artichoke) on glucose control, lipid profile, oxidative stress, inflammation, and cardiorespiratory fitness in T2DM patients. Methods: A total of 60 T2DM patients were randomly assigned to receive antioxidant/prebiotic (supplement group, SG) or maltodextrin (control group, CG), (two capsules (350 mg)/meal after three meals and before bedtime, 2.8 g/day), for 60 days. Venous blood samples were collected at baseline and after 60 days intervention to assess blood metabolic variables (glucose, insulin, and lipid profiles, renal and liver functions, oxidative stress, inflammation). Nutrition status, anthropometry, body composition (DEXA) and cardiorespiratory fitness were also measured. Results: Analysis of co-variance showed superior effects on T2DM’s glucose and lipid profiles in the SG compared with the CG including reduced fasting blood glucose (*p* = 0.01 within-group effects, *p* = 0.03 interaction effects), reduced glycated haemoglobin (*p* = 0.004 within-group effects, *p* = 0.002 interaction), and reduced low density lipoprotein (*p* = 0.006 within-group effects, *p* = 0.02 interaction effects). No significant change was found within the CG for any of these parameters. Kidney function’s glomerular filtration rate was also improved in the SG (*p* = 0.01 within-group effects), but not in the placebo CG. Intermediatory biomarkers of oxidative stress, inflammation, and cardiorespiratory fitness were not significantly affected in either group with no interaction effects. No adverse effects were detected following the 60-day supplementation intervention. Conclusions: The findings suggest that a combined anthocyanin-fibre may be promoted as an adjacent therapy in patients with T2DM, but the intermediary mechanisms of action require further research.

## 1. Introduction

Type 2 diabetes mellitus (T2DM) is projected to affect more than three-quarters of a billion people worldwide by 2045, which increases associated healthcare burdens [1]. There is also an increase in T2DM complications, which are often characterised by the coexistence of hyperglycaemia and dyslipidaemia and increased risk of cardiovascular disease among T2DM patients [2]. Fortunately, T2DM and associated cardiovascular risks can be prevented by lifestyle involving nutrition and physical activity approaches [3]. Large cohort studies have demonstrated superior effectiveness of lifestyle interventions on T2DM outcomes compared with standard glucose-lowering medications [4,5,6]. Therefore, developing novel nutritional therapeutics for improving glycaemia and lipidaemia T2DM patients is essential alongside standard treatments.

Functional foods are nutrients with beneficial biochemical properties, that have been shown to enhance T2DM primary outcomes of improved glycated haemoglobin (HbA1c), insulin sensitivity, glycaemic control, and secondary outcomes of antioxidant, anti-inflammatory, and anti-cholesterol functions [7]. For example, consuming fruits, vegetables, and whole grains provides T2DM patients with protective nutraceutical properties from phenolic compounds, terpenoids, flavonoids, alkaloids, sterols, and pigments [7].

Recent research attention has promoted anthocyanin, which is a plant flavonoid present in pigments of fruits, vegetables, and some whole grains, due to its antioxidant, anti-inflammatory, anti-diabetic, pro-vascular, and gut microbiota modulation properties [8,9]. Recent systematic reviews on T2DM patients concluded that anthocyanin supplementation for 4 weeks or over is effective in reducing HbA1c, FPG, 2 h postprandial glucose, triglycerides (TG), and low-density lipoprotein-cholesterol (LDL-C) [9,10]. Anthocyanin is also effective in reducing T2DM complications through modulating endothelial function, uric acid levels, and microbial metabolites in both prediabetes and diabetes patients [11]. Anthocyanin can induce better T2DM and cardiovascular outcomes, if consumed in combination with gut modulating prebiotics that enhance its cardiometabolic functions [9,10,11]. Prebiotics are abundant in dietary fibre, which is classified as soluble and insoluble fibre. Dietary fibre is defined by the American Association of Cereal Chemists as the edible portion of carbohydrates that are resistant to digestion and adsorption in the small intestine with partial or complete fermentation in the large intestine, and can be extracted from various cereals [12]. Among soluble dietary fibre, rice bran, and inulin have been receiving a lot of attention recently, especially in Asia where rice is widely consumed and produced agriculturally. For example, in Thailand, inulin from Jerusalem artichoke and rice bran from white rice (*Oryza sativa* L., RD6) are considered national agricultural products. An intervention study showed that rice bran and its fractions (water soluble and fibre concentrate) fed to 31 individuals with T2DM for 60 days reduced HbA1c by 15% and fasting glucose by 33%, and increased serum insulin by 4% [13]. Antihypertensive cardiovascular benefits were shown when rice bran extracts were supplemented daily for 12 weeks in patients with grade 1 hypertension, suggesting enhanced vasodilation-mediated endothelial mechanisms [14]. When white rice supplementation was compared with prebiotics and anthocyanin-rich grains such as riceberry rice, better glycaemic control (indicated by delayed postprandial glucose absorption and reduced gastric emptying) was found in healthy humans [15]. Rice bran and riceberry rice are commonly consumed in many Asian countries. A hypoglycaemic effects were attributed to improved intestinal microbiota [15] or glucose-dependent insulinotropic polypeptide (GIP) concentrations [16]. Furthermore, systematic reviews reported that inulin doses of up to 20 g/day, supplemented for 2–12 weeks in T2DM patients, decreased glucose and lipid parameters including TC, LDL-C, and TG [17,18]. In experimental articles, T2DM patients who received 10 g/day inulin for 8 weeks provided favourable effects on glycaemic status and lipid profiles in women with T2DM [19]. Comparing with rice bran extract, inulin extract exhibited lower prebiotic properties, displaying less effective stimulation of probiotics [20]. The mechanisms in which inulin consumption modifies T2DM glucose and lipid profile outcomes are inconclusive. However, it is likely that those are linked with improved insulin sensitivity [17,18], intestinal microbiota [21], or GIP concentrations [16]. Interestingly, combining anthocyanins from riceberry rice with prebiotics from rice bran (*Oryza sativa* L., RD6) and inulin fibre (Jerusalem artichoke) has been shown to significantly improve FPG and lipid profile in individuals with obesity [22]. No studies have yet explored whether and how supplementing such anthocyanin-prebiotic combinations would affect T2DM outcomes and associated cardiovascular risks in patients living with T2DM.

We hypothesised that a combined anthocyanin antioxidant (riceberry rice extracts) with prebiotics (rice bran and inulin fibre from Jerusalem artichoke) would enhance primary T2DM outcomes of HbA1c, insulin and glucose control, and secondary cardiovascular outcomes of lipid profile, oxidative stress, inflammation, and cardiorespiratory fitness in patients with T2DM.

## 2. Materials and Methods

### 2.1. Study Design

The study design is a clinical randomised, placebo-controlled, double-blinded, pre- and post-test intervention trial. The study was approved by the Human Ethical Committee of Khon Kaen University (HE621292) and followed the 2013 Declaration of Helsinki for testing human participants. The study was registered with the Thai Clinical Trials Registry (TCTR20190804003) on 4 August 2019. All study participants provided their informed written consent and received a full explanation of the purpose of the research and the experimental procedures in writing and verbally from the researchers.

**Figure 1:**
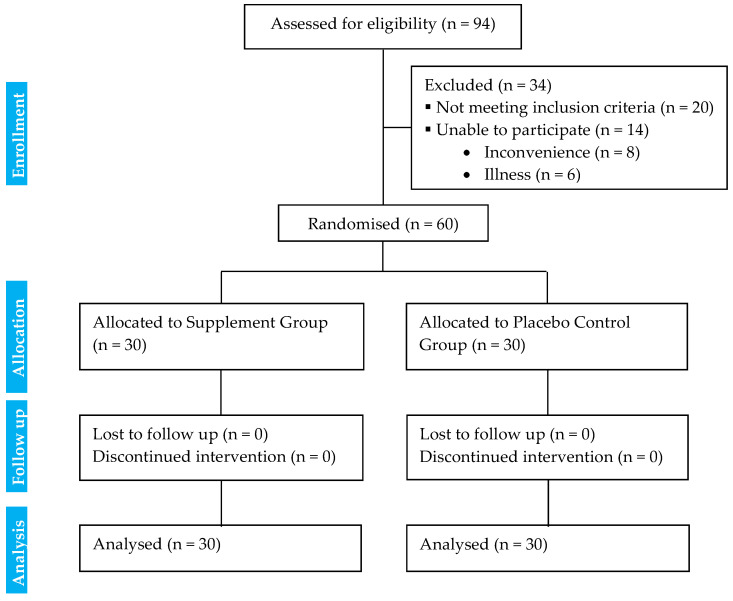
CONSORT flow diagram of the study.

The study recruitment followed Consolidated Standards of Reporting Trials (CONSORT) [23], (Figure 1), and a CONSORT checklist is provided in Supplement S1, Table S1. The study intervention trial lasted for 60 days from 9 December 2019 to 2 January 2021 and involved all participants performing two separate assessments conducted at baseline (pre-assessments) and the end of the 60-day intervention (post-assessments). The intervention and the safety analysis were based on a modified intention-to-treat principle (full analysis set). All data were collected at the Exercise and Nutrition Laboratory, Khon Kaen University. All tests were performed at similar laboratory environmental conditions (room temperature was 25 ± 2 °C, and the humidity was 46 ± 7%).

### 2.2. Study Population

#### 2.2.1. Sample Size Calculation

The sample size was calculated to achieve a reduction in FPG concentration in the supplement group of 28.7 mg/dL in FPG to meet the clinically meaningful difference based on previous research with a similar supplement ingredient (used inulin) [24]. To obtain a large effect size (Cohen’s d = 0.72) [25], and to achieve 80% power at an alpha level of 0.05, the statistical calculations required 60 patients to participate in this study, including a 20% dropout rate.

The study recruited 94 participants through advertisements on city and university websites and by word of mouth, and all participants were residing in Khon Kaen Province, Thailand. Sixty patients (56 females and 4 males) were diagnosed with T2DM out of the initial 94 patients (88 females and 6 males) who were initially recruited and screened, following the inclusion criteria (Figure 1).

#### 2.2.2. Inclusion and Exclusion Criteria

Participants were included if they aged between 30 and 60 years and were confirmed by their endocrinologist physician as patients diagnosed with T2DM (FPG ≥ 126 mg/dL) based on The American Diabetes Association (ADA) diagnosis guidelines 2011 [26] for longer than 6 months prior to the study’s start. All participants were receiving oral hypoglycaemic agent medication and had a normal lipid profile (<150 TG mg/dL, <200 total cholesterol (TC) mg/dL, ≥50 high-density lipoprotein cholesterol (HDL-C) mg/dL, <100 LDL-C mg/dL, and ≤2 TG/HDL-C) or had dyslipidaemia with the lipid-lowering drug, which they maintained throughout the intervention, had normal BP (systolic blood pressure (SBP) < 140 mmHg, or diastolic blood pressure (DBP) < 90 mmHg) or received an antihypertensive drug, which they maintained throughout the intervention. Full participants’ medication details were recorded (Supplement S1, Table S2). In terms of participants’ lifestyles, all participants are sedentary based on self-reported physical activity levels of less than two sessions per week of moderate or heavy intensity. All participants reported no alcohol consumption or smoking. Participants’ lifestyle behaviours were obtained using a lifestyle health questionnaire embedding questions on physical activity, dietary, smoking, and alcohol consumption habits (Supplement S2).

Patients were excluded (a) if they were reported to be allergic to rice or Jerusalem artichoke intake, which was checked and confirmed both verbally and in writing using a dietary questionnaire; (b) if they had experienced adverse reactions to having blood drawn or have haemophilia; (c) if they were diagnosed with any infection; (d) if they had other diagnosis in addition to T2DM including liver, kidney, respiratory tract, cardiovascular, neuromuscular, gastrointestinal, or other endocrine diseases; (e) if they did not intend to maintain medications for T2DM, dyslipidaemia, or hypertension; (f) if they regularly consumed nutritional or hormonal supplements in the last 3 months preceding the intervention.

#### 2.2.3. Randomisation, Allocation, and Blinding

We used blocked randomization to assign the patients into 2 equal blocks (*n* = 30 in each block) at a 1:1 ratio using a computer-generated randomization method, using the Research Randomizer [27], and patients were assigned to the supplement group (SG) or control group (CG) using their unsorted identification codes. The codes were in the same colour and size opaque envelopes. The capsules were coded by a researcher at the Faculty of Food Technology and the codes were sent to the technician who gave the capsules to participants. All researchers, data collectors, outcome adjudicators, data analysts, and patients were blinded to the intervention codes until the study was completed.

#### 2.2.4. Supplements Safety, Storage, and Preparation

##### Safety Testing

The supplements and placebo capsules were prepared under good manufacturing practises at the Department of Food Technology, Faculty of Technology, Khon Kean University, Thailand [22]. The whole supplementation batch used for the intervention (3 kg) was declared an acceptable value as a standard requirement of the Thai Food and Drug Administration for this intervention by Toxic Substance, Contaminant and Dioxin Section, Bureau of Quality and Safety of Food, Department of Medical Sciences, Ministry of Public Health the laboratory of the National Food Institute, Ministry of Industry, Thailand [28]. The tests included Aflatoxin, bacteria (aerobic plate count, *Clostridium perfringens*, *Staphylococcus aureus*, *Escherichia coli*, and *Salmonella* spp.), and heavy metals (lead, arsenic, and mercury).

##### Preparation, Content, and Storage

The supplement and the placebo capsules were prepared as individual 350 mg capsules [22] of similar shape, taste, size, colour, odour, and appearance. The supplement composition is 1.26 g/day of each dietary fibre (from Jerusalem artichoke and white rice bran) and 0.28 g/day of anthocyanin (from riceberry rice). Their chemical composition amounts and analytical methods: soluble fibre [29,30], inulin [29,30], anthocyanin [31,32], antioxidant activity [31,33] and were analysed by the Department of Food Technology, Faculty of Technology, Khon Kean University, Thailand, as shown in Supplement S1, Table S3. The placebo contained maltodextrin (Zhucheng Dongxiao Biotechnology Co., Ltd., Zhucheng, China). All capsules were kept in opaque bags labelled with codes, expiration dates, and ingestion details, and were stored at 4 °C.

### 2.3. Experimental Procedures

This intervention trial involved a 60-day supplementation with pre- and post-assessments of T2DM outcomes using a combination of anthropometry and body composition, blood sampling for metabolic outcomes, lifestyle nutrition and physical activity habits, cardiorespiratory fitness, and clinical safety for adverse effects. Pre- and post-assessments of all participants were performed at the outpatient Diabetes Clinic in Community, Faculty of Medicine, Khon Kaen University. All participants reported to the clinic at 7:00 a.m. following a 12-h fast and refraining from alcohol, caffeine, and strenuous exercise. The same trained personnel consistently performed all pre- and post-assessments of anthropometry, body composition, and haemodynamic measurements.

Prior to baseline (pre-intervention) assessments, all participants received diaries to record a three-day diet and physical activity (Table 1). These diaries were used to ensure all participants had maintained their daily regular dietary and physical activity habits using validated questionnaires of Food Frequency Questionnaire (FFQ) [34] and International Physical Activity Questionnaire (IPAQ) [35]. FFQ was analysed for macronutrients (carbohydrate, fat, and protein) and micronutrients using the INMUCAL program (INMUCAL software, version 4.0, Mahidol University, Nakhon Pathom, Thailand), and IPAQ by converting daily activity levels into the metabolic equivalent (MET) and corresponding energy expenditure according to the compendium of physical activities [36], (Equation (1)):

Energy Expenditure (kcal) = MET (kcal·kg^−1^ h^−1^) × body weight (kg) × time (h) (1)

#### 2.3.1. Pre-Intervention Assessments

##### Anthropometry and Body Composition Measurements

Body mass and height were measured using a stadiometer (DETECTO, Webb City, MO, USA) in a standing position with the participants minimally clothed and without shoes. Both parameters were used to calculate the body mass index (BMI, kg/m^2^). Waist circumference was measured at the midpoint between the lower rib margin and the iliac crest at the end of normal expiration, with an inelastic tape measure per the WHO guidelines [37]. Hip circumference was measured at the widest point. Dual-energy X-ray absorptiometry (Lunar Prodigy whole-body scanner, GE Healthcare, Madison, WI, USA) was used to measure the total body composition, lean body mass, and fat mass in lying supine and wearing light indoor clothing.

##### Heart Rate and Blood Pressure Measurements

The participants’ HR and BP were measured at the brachial artery after 30 min of resting quietly in a supine lying. The BP was measured using an automatic sphygmomanometer (UA-767 Plus, Abingdon, Oxfordshire, UK) with the cuff wrapped around the subjects’ upper right arm, in a sitting position, and measurements of HR and BP were repeated three times at 2 min intervals, and the average of the last two measures was recorded.

##### Supplementation Protocol

All participants were assigned to receive one of two capsules of the supplement (SG) or the matching placebo capsule (CG), both taken daily as two capsules after their three meals and before bed for 60 days. Participants were provided with a box containing the total number of the supplement capsules in two halves, one half at day one and the remaining half at day 30. The box contained matching supplement capsules of SG and the placebo CG. The box was of a similar appearance and weight, and the capsules inside were of a similar appearance, weight colour, and odour.

**Figure 2:**
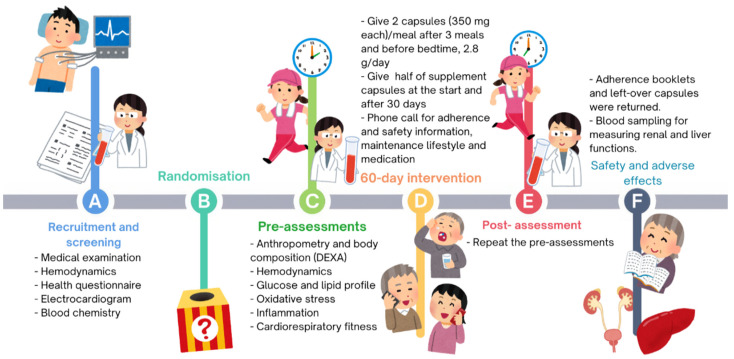
Study intervention protocol. Abbreviations: DEXA, dual-energy X-ray absorptiometry.

Participants also received record form to tick for each daily intake of capsules and any missed days (Supplement S1, Table S4). Monitoring of supplementation adherence throughout the intervention was performed through weekly telephone calls from the same technician. Participants were asked to return any unused supplements at the end of the 60 days during their last re-assessment visit. All intervention and experimental procedures are schematically displayed in Figure 2 below:

##### Blood Sampling and Analysis Protocol

- Blood Sampling

Blood was drawn from the antecubital veins with the participant at rest for at least 5 min and in a supine position. The collected blood samples were immediately separated into four tubes: 1 mL blood in a sodium fluoride tube to measure glucose concentration; 9 mL blood in ethylenediaminetetraacetic acid (EDTA) tube to measure lipid profile, HbA1c, white blood cells (WBC) count, malondialdehyde (MDA), and Cr concentrations; 3 mL blood in a gel and clot activator tube to measure the serum concentrations of serum glutamic-pyruvic transaminase (SGPT), high sensitivity C-reactive protein (hsCRP), and insulin; 3 mL blood in a lithium heparin tube wrapped in aluminium foil was used to provide a plasma aliquot (500 µL) by adding 500 µL perchloric acid for protein precipitation to measure the plasma vitamin C concentration. All the tubes except the sodium fluoride tube were then centrifuged at 3000× *g* (TOMY-CAX-370, Tokyo, Japan) for 10 min at 4 °C. The upper layer was then immediately transferred into aliquots, which were frozen at −80 °C until their analyses.

- Analysis of Blood Glucose and Lipid Profile

FPG concentration was analysed for plasma by a glucose analyser (YSI 2300 STAT Plus™, YSI, Yellow Springs, OH, USA) using an enzyme-based biosensor to measure glucose concentrations. A 25 µL sample from a sodium fluoride tube (Greiner Bio-One Ltd., Chonburi, Thailand) was pumped into the analyser and then diffused through the membrane, containing an immobilised glucose oxidase enzyme, which was rapidly oxidised, producing hydrogen peroxide, which in turn oxidises at the platinum anode, producing electrons. The electron flow is linearly proportional to the steady-state hydrogen peroxide concentration and, therefore, to the blood glucose concentration.

HbA1c was analysed by an immuno-turbidimetric inhibition assay at the Biochemistry Laboratory of the Srinagarind Hospital. Plasma insulin concentrations were assayed with a radioimmunoassay kit (MP Biomedical GmbH, Eschwege, Germany) at Srinagarind Hospital, Faculty of Medicine, Khon Kaen University. Fasting glucose and insulin concentrations were then used to calculate insulin resistance (homeostatic model assessment for insulin resistance, HOMA-IR [38], (Equation (2))

HOMA-IR = [fasting Insulin (μg/mL)] * [fasting Glucose (mmol/L)]/22.5(2)

Triglyceride, HDL-C, and TC concentrations were analysed using the glycerol phosphate oxidase-phenol 4-aminoantipyrine peroxidase (GPO-PAP) method, the homogeneous HDL-C plus method, and the cholesterol oxidase-peroxidase method, respectively (Reflotron^®^Plus (Boehringer Mannheim, Mannheim, Germany), and the machine was calibrated daily. LDL-C concentrations were calculated using the Friedewald formula [39].

- Analysis of Markers of Oxidative Stress and Inflammation

Plasma MDA concentrations were determined using Draper’s method [40]. In brief, the method involved mixing 150 µL of plasma with 125 µL of 10% TCA (Aldrich Chemis-try, Darmstadt, Germany), 125 µL of 5 mM EDTA (Sigma life Science, Darmstadt, Germa-ny), 125 µL of 8% Sodium Dodecyl Sulphate (SDS) (Sigma life Science, Darmstadt, Germany), and 10 µL of 0.5 µg/mL Butylated hydroxytoluene (BHT) (Aldrich Chemistry, Darmstadt, Germany), respectively. Samples were then incubated for 10 min at room temperature and were added 125 µL of 0.6% Thiobarbituric Acid (TBA) (Sigma-Aldrich, Steinheim, Germany). The mixture was then boiled in a water bath for 30 min and then cooled down. After cooling down, the mixture was centrifuged at 10,000× *g* rpm for 5 min. The absorbance of the supernatant at 532 nm was then measured using a spectrophotometer.

Plasma vitamin C concentration was assessed using Zhang’s method [41]. In short, 100 µL of each deproteinised plasma with 100 µL of FeCl3 (Panreac AppliChem ITW com-panies, Darmstadt, Germany) and 100 µL of K3Fe[CN]6 (Ajax Finechem Pty Ltd., Taren Point, Australia) were added. Then, 2200 µL of distilled water to the solution was added to adjust to 2500 µL following by incubating at 20° in an ice bath for 30 min. A spectro-photometer was then used to measure the supernatant at 735 nm. Protection from light and ice was ensured at every step of the assay.

The WBC count and hsCRP concentration were used to determine general inflammation, which was performed at laboratories of Srinagarind Hospital, Khon Kaen University, Khon Kaen, Thailand.

- Analysis of Markers of Renal and Liver Functions

Blood parameters included renal function indicators of serum creatinine (Cr) and estimated glomerular filtration rate (eGFR), and liver function enzyme (SGPT). The plasma Cr and SGPT concentrations were measured with the creatinine iminohydrolase–peroxidase method and the pyruvate oxidase–peroxidase method, respectively (Reflotron Plus: Roche, Boehringer Mannheim, Mannheim, Germany). The eGFR was calculated using the Cr-based chronic kidney disease epidemiology collaboration (CKD-EPI) equation [42].

All blood parameters, including glucose, insulin, lipid profile, vitamin C (antioxidant), MDA, WBC, hsCRP, Cr, and SGPT, were tested using duplicates, and then averaged values were calculated. Their thresholds are shown in Supplement S1, Table S5.

##### Cardiorespiratory Fitness Assessment

The cardiorespiratory fitness (VO_2peak_) was estimated using the 6-minute walk test (6MWT) [43].

The testing procedures followed ACSM’s recommendation for T2DM patients [43]. It involved each participant walking continuously for 6 min as fast as possible, without jogging or running, and with verbal prompts for encouragement every minute until reaching the 6th minute [44]. The highest distance value in metres was recorded, while incomplete steps were discounted. All tests were performed at a similar time of the day and by the same technician. VO_2peak_ was calculated from the distance covered (Equation (3)).

VO_2peak_ = 14.986 + (0.025 × walk distance) − (0.161 × body weight). (3)

#### 2.3.2. 60-Day Intervention

During the intervention, all participants were followed once per week, every week with a 20-minute phone call by the same researcher (C.T.), which involved feedback on completing the supplement adherence booklet, adherence, and questions on any side effects. Each missed supplementation was recorded and returned at the end of the study (Supplement S1, Table S6).

Patients received half of the supplement capsules at the start of the intervention and made an additional visit to the clinical laboratory after 30 days to receive the remaining half of the supplement capsules. During this intermediary visit, patients received a gratuity monetary reward for their participation and were further briefed about the adherence.

Throughout the intervention, participants were asked not to change their daily physical activity or dietary habits (e.g., start any additional exercise or ingest new foods rich in prebiotics or antioxidants, especially artichoke or berries). The patients consumed typical Thai cuisine, which has many healthy antioxidants and prebiotic ingredients, but they reported no significant changes to their dietary habits (Table 1).

All patients maintained their regular contact with their physician throughout the intervention without any change, contraindications, or a change to their regular medications due to their participation in this study. None of the patients reported any adverse effects during the 60-day intervention period. Furthermore, no other side effects were reported concerning the menstrual cycle (See patient characteristics, Table 2).

#### 2.3.3. Post-Intervention Assessments

All pre-assessments were repeated using the same procedures for anthropometry, body composition, heart rate, blood pressure, glucose and lipid profiles, fitness, and lifestyle habits. Additionally, patients were asked about any post-intervention adverse effects. All adherence booklets and left-over capsules were returned to the laboratory during the last visit. At the end of the intervention, all patients received the same gratuity monetary reward and were thanked for their participation.

### 2.4. Data Analysis and Statistics

Normal distribution was tested with the Shapiro–Wilk Test. The distributions of blood glucose, plasma lipid profile, plasma MDA, plasma vitamin C, plasma hsCRP, 6MWD, and energy intake and expenditure were normal. The data were mean ± SE except where stated elsewhere. Individual independent differences between the groups at baseline were compared using independent *t*-test. The supplementation effects were analysed using one-way ANCOVA. The data that were not normally distributed of serum hsCRP, serum insulin, and HOMA-IR were presented as median (IQR), and compared for within-group differences using Wilcoxon Signed rank test and for between-group differences using Mann–Whitney U test. All differences were considered significant at *p*-value < 0.05, and Confidence Intervals (CIs) were presented at 95%. All the statistical analyses were performed with SPSS version 27 (IBM, Armonk, NY, USA).

## 3. Results

All participants had T2DM with 80% of them had been living with diabetes for longer than 2 years (Table 2). The compliance rate with supplementation was 97.8% and 96.8% for SG and CG, respectively (Supplement S1, Table S6). No adverse effects of the supplement throughout the intervention were reported.

The two participant groups had similar nutrition status with no baseline differences (Table 1). The intervention did not affect nutrition status with no differences in macronutrients, micronutrients, energy intake and expenditure within and between groups, nor was there interaction effects (Table 1). The demographic, anthropometric, and body composition characteristics of the two randomised groups CG and SG were not different at baseline (all *p* > 0.05, Table 2). There were no intervention effects in any of the groups nor was there an interaction effects (duration × supplement) for any of those characteristics (all *p* > 0.05, Table 2).

### 3.1. Diabetes Biomarkers

#### 3.1.1. Glucose and Lipid Profiles

At baseline, there was no difference between the groups for any of the glucose or lipid profiles. However, following the 60-day supplementation intervention, an improvement in diabetes and lipid profile outcomes of FPG, HbA1c, and LDL-C were only found within the SG but not within the CG (Table 3). The ANCOVA analysis also showed superior effects within SG to those in CG for reducing FPG (*p* = 0.01 within-group effects, *p* = 0.03 interaction effects), HbA1c (*p* = 0.004 within-group effects, *p* = 0.002 interaction), and LDL-C (*p* = 0.006 within-group effects, *p* = 0.02 interaction effects), (Table 3). TC was also reduced in SG but not in CG (*p* = 0.002 within-group effects). Additionally, renal function indicated by eGFR was improved within the SG but not within the CG (Table 3). There were no significant differences for any of the remaining variables as shown in Table 3, and Supplementary File Figures S1 and S2).

#### 3.1.2. Oxidative Stress and Inflammation Blood Biomarkers

After supplementation, the plasma MDA, ascorbate, and hsCRP concentrations and WBC counts did not significantly differ within or between groups and were maintained within a normal healthy range (Table 4) in the CG. Although there were no significant differences in hsCRP and WBC counts, they decreased after the SG supplementation but did not change in the CG. The post-supplementation of both variables tended to be lower in the SG than the CG (*p* = 0.07).

#### 3.1.3. Cardiorespiratory Fitness

Cardiorespiratory fitness measured by 6MWT was not significantly different within or between groups (Table 5).

#### 3.1.4. Adverse Effects

There were no adverse symptoms such as flatus belching, bloating, nausea, abdominal cramping, a feeling of fullness, stomach upset headache, fatigue, vomiting, diarrhoea, or cardiac dysrhythmia from any of the supplements. Neither group showed any adverse changes in liver or kidney functions as indicated by no significant changes in plasma Cr or serum SGPT, and a positive increase in eGFR concentrations (Table 3).

## 4. Discussion

The main finding of the present study is that T2DM outcomes were improved following the supplementation with a blend of anthocyanin antioxidants (riceberry rice extracts of *Oryza sativa* L.) with prebiotics (inulin fibre from Jerusalem artichoke and white rice bran). This 60-day clinical trial involved a large group of T2DM sedentary patients and resulted in significant reduction in patients’ HbA1c, FPG, and LDL-C in the SG but not in the CG, and with superior effects in the SG than in the CG. These improvements in glucose control and lipid profile outcomes in T2DM patients promote our supplement of a combined anthocyanin antioxidants with prebiotics inulin fibre and rice bran as an effective adjacent therapeutic treatment for T2DM.

The study’s glucose- and lipid-lowering supplementation benefits reflected an 11.91%, 6.71%, 8.39%, and 13.36% reduction in FPG, HbA1c, and LDL-C, respectively, which are considered clinically meaningful in T2DM treatment and are in line with previous studies, which used a single prebiotic or antioxidant ingredient supplement [13,24,45,46]. This study used a much lower dose of a combined blend (0.021 ± 0.004 mg/kgBM/day) of each prebiotic fibre with 0.28 g/day (0.005 ± 0.001 mg/kgBM/day) anthocyanin from riceberry rice). In contrast, Li et al. (2015) supplemented a higher dose of 320 mg/day (0.32 g/day) anthocyanin (bilberries and blackcurrants extracts) for 24 weeks in T2DM patients, which resulted in an 8.5% lower FPG, 13% lower HOMA-IR, 7.9% lower LDL-C, 23% lower TG, and 19.4% higher HDL-C [45]. Another intervention in T2DM with a similar duration to our study (around 60 days), reported better improved glucose and lipid profiles (reduced FPG, HbA1c, TC, and LDL-C of 33%, 15%, 9.2%, and 13.7%, respectively) when a higher dose (0.6 g/kgBM/day) of stabilised rice bran concentrate was combined with a healthier AHA Step-1 Diet [13]. Cheng and coworkers (2010) used almost double our dose of rice bran (2.0 g/day vs. 1.2 g/day) with a longer duration intervention (24 weeks vs. 60 days) to induce a 10–15% reduction in postprandial glucose and HbA1c levels in T2DM patients [46]. Increasing the inulin doses for up to 20 g/day, supplemented for 2–12 weeks in T2DM patients has been suggested to induce a marked reduction in glucose and lipid parameters in both men and women with type 2 diabetes [17,18,19,47]. The latter studies collectively agree that a moderate dose of prebiotic rice bran fibre and a high dose of inulin supplementation is considered effective as complementary T2DM therapeutic, but our study added the antioxidant benefit of anthocyanin as additional functional food beneficial supplement using relatively low doses. Functional foods are well established for managing T2DM and preventing its complications, especially when combined with active lifestyle approaches [7,48].

Our study combined anthocyanin antioxidant with prebiotic inulin from Jerusalem artichoke, which has never been chronically supplemented before in T2DM patients. Previous limited research on inulin pure extract in T2DM patients did not provide the inulin source details in T2DM and administered a much higher inulin dose (130 mg/kgBM/day or 10 g/day), which was almost five-fold higher than our inulin dose (21 mg/kgBM/day) for a similar 2-month duration [24]. The study reported a decreased FPG (8.47%) and HbA1c% (10.43%) levels, which is close to our reported change (Table 3), suggesting that our study’s artichoke inulin fibre is an effective prebiotic source for preventing and managing T2DM. Another study in hypercholesterolemic participants who took 18 g/d of inulin in a low-fat diet for 6 weeks showed a reduction in LDL-C (14.4%) and TC (8.7%) [49]. In comparison, this study showed improvements in FPG (11.91%), %HbA1c (6.71%), and LDL-C (13.36%) (Table 3), suggesting that a lower dose of our supplement can be effectively and efficiently administered to induce such benefits in T2DM patients.

This study did not find significant effects on oxidative stress biomarkers indicated by levels of lipid peroxide and vitamin C levels in T2DM patients following the 60-day supplementation of antioxidant/prebiotic blend (Table 4). It is likely that the prebiotic and probiotic ingredients (e.g., anthocyanins, inulin fibre) did not result in a synergetic antioxidation effects. Such synergy of “synbiotics” is a matter of recent research debate and may work differently in different patient groups [50]. For example, in participants with overweight and obesity, a similar supplement blend was inferred to be an effective in raising plasma vitamin C level following a shorter 4-week supplementation intervention [51]. Another explanation is the severity of T2DM in patients of this study may explain the lack of effectiveness on antioxidation markers. Participants of this study had been diagnosed with T2DM for longer than 2 years and had obesity complications, which may explain a potential renal leak of ascorbate, often associated with impaired FPG and HbA1c levels [52]. An improved kidney function indicated by eGFR in the SG (91 to 95 mL/min/1.73 m^2^) compared with non-significant increase in CG (vs. 93 to 94 mL/min/1.73 m^2^), (Table 3), suggests that a higher dose of an antioxidant anthocyanin may augment the postulated synbiotic effectiveness in individuals with advanced T2DM patients [53]. Previous studies in patients with T2DM increased antioxidant activity indicated by ferric ion reducing antioxidant power by 29.8% following 320 mg of anthocyanin/day supplementation for 24 weeks [45]. Another study found the intake of 10 g/day inulin for 60 days attenuated hs-CRP [19]. Of note, our study’s supplement tended to decrease inflammation, as shown by reduction in hsCRP and WBC counts (*p* = 0.07). Accordingly, future studies should consider a greater dose of anthocyanin and inulin to induce significant beneficial effects on the antioxidant and inflammatory activities.

Perhaps, this study’s hypoglycaemic mechanisms can be explained using other biochemical routes than anti-inflammation, including anthocyanin’s inhibitory mechanisms on alpha-glucosidase and alpha-amylase, which decrease intestinal glucose absorption [54]. Moreover, Jerusalem artichoke inulin has a hypoglycaemic effect via improved intestinal microbiota [21] or glucose-dependent insulinotropic polypeptide (GIP) concentrations [16]. The former produces short-chain fatty acids (SCFAs), which have been shown to regulate FPG concentrations through various mechanisms [55]. The latter [56] is an intestinal hormone with many physiological actions. In the postprandial state, GIP stimulates insulin after a meal and glucagon secretion during eu- and hypoglycaemia. Other mechanisms can be related to rice bran’s improved glycaemic control by activating the IRS1/AKT/GLUT4 insulin signalling pathway leading to increased insulin sensitivity in skeletal muscle [57]. Nonetheless, insulin sensitivity was not significantly different in this study so the mechanisms stated can be accounted for collectively.

The lipid-lowering mechanisms found in this study (LDL-C, TG), are supported in both animal and human studies. Firstly, patients with T2DM who consumed 16 g of inulin-type fructans (a mixture of oligofructose and inulin) for 6 weeks had a significant bifidogenic effect and increased concentrations of faecal SCFA without any changes in faecal microbial diversity [58]. However, in diabetic mice, the hypolipidemic effect of inulin was found to increase the expression of genes involved in lipids and cholesterol metabolism including microbiota growth, for example, *Prevotellaceae UCG-001*, *Parasutterella*, *Prevotellaceae UCG-003*, and *Dubosiella* [59]. SCFAs themselves or products of the fermentation with the microbiotas, especially butyrate, and propionate, can inhibit hepatic cholesterol synthesis, thereby decreasing cholesterol concentrations. This is confirmed by a study on hypercholesterolemia, which found a reduction in LDL-C by −14.4% by inulin supplementation [49]. Compared to the above studies, this study only found an improvement in LDL-C, which was a similar amount (13.36%). Secondly, dyslipidaemia participants who consumed 320 mg/day anthocyanin for 12 weeks had a reduction in LDL-C by 13.6% [60]. The authors reported that the mechanism for this result is increased cellular cholesterol efflux to serum, which correlates with decreased mass and activity of plasma cholesteryl ester transfer protein (CETP). Furthermore, the functions of rice bran on viscosity, glucose dialysis retardation index (GDRI), cation exchange capacity (CEC), and cholesterol and bile salt adsorption capacity may be alternative mechanisms explaining the cholesterol-lowering effects, especially LDL-C [61]. It is noted that there has been no evidence of each ingredient of our antioxidant/prebiotic supplement in patients with T2DM. Thus, these suggested mechanisms need further confirmation in this study’s patients.

This study’s T2DM patients were all sedentary with reduced levels of cardiorespiratory capacity estimated using the 6-minute walk test. The 60-day intervention supplementation did not significantly affect patients’ VO_2peak_ (15–16 mL/kg/min) or the distance covered in the 6-minute test (Table 5). Although this intervention did not postulate to change patients’ physical activity behaviour, a sedentary lifestyle in T2DM remains a complex issue requiring a multi-component intervention [62,63]. Adding physical activity training is recommended to ameliorate sedentary related risks in T2DM patients, especially in optimising the intestinal microbiota composition [64]. Exercise training also augments the supplementation effectiveness, though this should form part of a personalised lifestyle programme that considers patients baseline fitness, medication, and nutritional supplementation [6]. Furthermore, no adverse effects of multiple supplements on liver or kidney function were observed in this study. Accordingly, our multiple supplements may be used for diabetes drug therapy without any possible interactions. Despite having antihyperglycaemic and antilipidaemic effects and an anti-inflammatory trend, either the dose or the duration of supplementation may be insufficient to meet the significant differences in other variables, including other lipid profile (plasma TC, TG, and HDL-C), insulin resistance, oxidative stress, inflammation, and aerobic capacity.

This double-blinded randomised clinical trial relied on a robust methodology in line with the best randomised controlled trial practice that ensures validity and reliability [65]. It has a major strength in achieving a very high compliance rate (97.8%) in patients with type 2 diabetes after 60 days of supplementation, with no reported adverse effects. However, a limitation is that even though our inclusion criteria had been open to men and women, the majority of our T2DM participants were women, who adhered very well and were of a similar ethnicity. In Thailand, healthy attitudes towards lifestyle interventions are known. High frequency of attendance was shown not to be related to improved blood parameters in Thai patients with prediabetes and hypertension [66]. The latter reported that the knowledge obtained from program intervention together with adequate contact between the patient and the program staff, personal problems solving, personal attitude, and practice played a key role for long-term successful lifestyle modifications. This type of intervention engagement may explain the no between-groups difference found in blood profiles, explained by some improvements (though not-significant) found in the placebo CG. Nonetheless, our supplementation intervention was effective in lowering glucose and lipid levels in the SG, compared with the placebo CG who did not have those effects, with significant within-group and interaction effects. The results support the practical application of our supplement as a safe and effective adjuvant treatment for type 2 diabetes.

## 5. Conclusions

This study suggests diabetes therapeutic benefits of the intake of a combined prebiotic/antioxidant supplement blend containing low doses of inulin, anthocyanin, and rice bran, in patients with type 2 diabetes. Improved glycaemia and lipidaemia profiles were found following 60-day supplementation regardless of physical fitness, suggesting the effectiveness of this supplement blend as an adjacent T2DM therapy with no adverse effects. Future studies should consider higher doses, especially anthocyanin, to induce further effects on the oxidative stress and inflammation profiles. Adding physical activity training and longer duration of intake of functional foods containing prebiotics and antioxidants is likely to augment its diabetes-preventative benefits.

## Figures and Tables

**Table 1:** Energy intake and expenditure of the participants before and after supplementations in both groups.

	CG			SG			Mean Difference [95% CI]	Between Groups at Baseline *p*-Value	Interaction Effects (Duration × Supplement) *p*-Value
	Before (*n* = 30)	After (*n* = 30)	Duration Effects (Within Group) *p*-Value	Before (*n* = 30)	After (*n* = 30)	Duration Effects (Within Group) *p*-Value			
Protein (g/day)	64 ± 3.8	64 ± 3.5	0.98	62 ± 3.0	72 ± 5.0	0.05	10 [−3.9, 20.8]	0.64	0.18
Carbohydrate (g/day)	169 ± 10.6	163 ± 10.9	0.59	170 ± 9.9	184 ± 8.2	0.16	20 [−3.5, 44.4]	0.93	0.09
Fat (g/day)	31 ± 2.8	31 ± 2.2	0.88	31 ± 2.4	36 ± 4.0	0.14	5 [−3.1, 14.0]	0.99	0.21
Vitamin C (mg/day)	68 ± 7.1	66 ± 6.2	0.77	67 ± 7.2	70 ± 6.4	0.70	5 [−12.5, 21.2]	0.91	0.61
Energy intake (kcal/day)	1211 ± 66.4	1185 ± 68.3	0.70	1298 ± 55.7	1294 ± 56.3	0.93	22 [−90.0, 206.4]	0.32	0.44
Energy expenditure (kcal/day)	1284 ± 62.4	1292 ± 61.6	0.85	1350 ± 59.2	1326 ± 60.2	0.57	−32 [−123.9, 88.9]	0.45	0.74

Data are expressed as mean ± SE (mean difference [95% CI], *p*-value); *n* = 60 (*n* = 30 each group, 28 females and 2 males). No significant differences in nutrition status were found at baseline between the groups, and no significant duration, supplement, or interaction effects were found (all *p* > 0.05). Abbreviations: CG: control group (with placebo supplement), SG: supplement group, CI: confidence intervals.

**Table 2:** Patients physical, socioeconomic, and demographic characteristics in both groups.

	CG			SG			Mean Difference [95% CI]	Between Groups at Baseline *p-*Value	Interaction Effects (Duration × Supplement) *p*-Value
	Before (*n* = 30)	After (*n* = 30)	Duration Effects (Within Group) *p*-Value	Before (*n* = 30)	After (*n* = 30)	Duration Effects (Within Group) *p*-Value			
Age ^$^ (yr)	53 ± 0.9			54 ± 0.9			1 [−3.4, 1.7]	0.5	
F/M (*n*)	28/2			28/2					
Diabetes duration ^$^ (yr)	7 ± 0.7			6 ± 0.8			−1 [−1.6, 2.5]	0.65	
Diabetes duration ^@^ (*n* (%))								0.49	
>2 years	24 (80.0)			26 (86.7)					
<2 years	6 (20.0)			4 (13.3)					
Menstruation ^@^ (*n* (%))								0.76	
Menstruation	8 (28.6)			7 (25.0)					
Menopause	20 (71.4)			21 (75.0)					
Marital status ^@^ (*n* (%))								0.50	
Married	29 (96.7)			28 (93.3)					
Single	1 (3.3)			1 (3.3)					
Widowed	0 (0.0)			1 (3.3)					
Occupation ^@^ (*n* (%))								0.65	
Merchant	7 (23.3)			4 (13.3)					
Farmer	9 (30.0)			9 (30.0)					
Employee	11 (36.7)			15 (50.0)					
Unemployed	3 (10.0)			2 (6.7)					
Education level ^@^ (*n* (%))								1.00	
<high school	28 (93.3)			28 (93.3)					
≥high school	2 (6.7)			2 (6.7)					
BM ^$^ (kg)	63 ± 2.1	62 ± 2.0	0.09	62 ± 2.0	62 ± 2.0	0.76	1 [−0.3, 1.8]	0.71	0.15
BMI ^$^ (kg/m^2^)	27 ± 0.8	27 ± 0.8	0.11	26 ± 0.8	26 ± 0.8	0.73	0 [−0.2, 0.7]	0.44	0.24
Nutritional status ^@^ (*n* (%))			1.00			0.62		0.72	
Normal ^#^	5 (16.7)	5 (16.7)		6 (20.0)	4 (13.3)				
Overweight ^#^	5 (16.7)	5 (16.7)		7 (23.3)	10 (33.3)				
Obese ^#^	20 (66.7)	20 (66.7)		17 (56.7)	16 (53.3)				
W ^$^ (cm)	91 ± 1.8	90 ± 2.0	0.11	91 ± 2.0	90 ± 2.1	0.44	0 [−1.4, 2.7]	0.96	0.55
H ^$^ (cm)	99 ± 1.5	99 ± 1.7	0.64	99 ± 1.5	98 ± 1.5	0.04	−1 [−2.6, 0.1]	0.90	0.07
W/H ^$^ ratio	0.90 ± 0.0	0.90 ± 0.0	0.05	0.90 ± 0.0	0.90 ± 0.0	0.55	0 [−0.0, 0.0]	0.87	0.08
BF ^$^ (%)	34 ± 1.0	34 ± 1.0	0.08	33 ± 1.0	34 ± 1.0	0.50	1 [−0.2, 1.8]	0.51	0.11
FM ^$^ (kg)	22 ± 1.1	22 ± 1.1	0.08	21 ± 1.2	22 ± 1.1	0.50	1 [−0.1, 1.3]	0.61	0.10
LBM ^$^ (kg)	40 ± 1.3	40 ± 1.4	0.47	40 ± 1.1	40 ± 1.1	0.95	0 [−0.9, 0.5]	0.83	0.58

There were no differences in any body composition and anthropometric characteristics between the groups at baseline, and there was no intervention effects or interaction (duration × supplement) effects for any of those characteristics (all *p* > 0.05). Data are expressed as mean ± SE ^$^ and frequency (*n*, %) ^@^. Asia-Pacific cutoff points ^#^: underweight < 18.5; normal 18.5–22.9; overweight 23–24.9; obese ≥ 25. Abbreviations: CG, control group; SG, supplement group; F/M, female/male: BM, body mass; BMI, body mass index; W, waist circumference; H, hip circumference; W/H ratio, waist to hip circumference ratio; BF, body fat; FM, fat mass; LBM, lean body mass.

**Table 3:** Blood chemistry variables of the participants before and after supplementations in both groups.

	CG			SG			Mean Difference [95% CI]	Between Groups at Baseline *p*-Value	Interaction Effects (Duration × Supplement) *p*-Value
	Before (*n* = 30)	After (*n* = 30)	Duration Effects (Within Group) *p*-Value	Before (*n* = 30)	After (*n* = 30)	Duration Effects (Within Group) *p*-Value			
FPG ^$^ (mg/dL)	217.9 ± 13.1	220.7 ± 18.0	0.82	230.1 ± 16.3	187.8 ± 12.3	0.01	−45.1 [−76.1, −4.6]	0.56	0.03
HbA1c ^$^ (%)	8.6 ± 0.3	8.8 ± 0.3	0.35	8.6 ± 0.5	7.9 ± 0.4	0.004	−0.9 [−1.5, −0.3]	0.92	0.002
Insulin ^γ^ (µIU/mL)	5.5 (0.0–10.8)	6.0 (0.0–10.0)	0.43	4.0 (0.8–7.8)	4.5 (0.0–8.3)	0.53	0.4 [−1.5, 0.7]	0.81	0.46
HOMA-IR ^γ^	3.0 (0.2–6.2)	3.1 (0.0–5.9)	0.60	2.3 (0.4–5.0)	1.8 (0.0–4.5)	0.52	−0.6 [−1.3, 0.3]	0.70	0.20
LDL-C ^$^ (mg/dL)	101 ± 7.3	102 ± 6.6	0.86	96 ± 7.4	82 ± 7.0	0.006	−15 [−29.1, −2.8]	0.63	0.02
TC ^$^ (mg/dL)	187 ± 7.6	180 ± 8.1	0.12	174 ± 9.5	160 ± 8.5	0.002	−7 [−21.0, 3.6]	0.30	0.16
TG ^$^ (mg/dL)	179 ± 21.1	171 ± 8.1	0.66	164 ± 16.1	171 ± 16.2	0.69	15 [−35.7, 50.6]	0.58	0.73
HDL-C ^$^ (mg/dL)	43 ± 2.0	44 ± 1.9	0.45	44 ± 2.1	44 ± 2.6	0.70	−0.4 [−4.9, 4.1]	0.75	0.85
SGPT ^$^ (U/L)	18.11 ± 1.3	17.56 ± 1.3	0.48	18.91 ± 1.1	18.93 ± 1.4	0.98	0.57 [−1.6, 2.8]	0.64	0.58
Cr ^$^ (mg/dL)	0.77 ± 0.0	0.76 ± 0.0	0.35	0.79 ± 0.0	0.76 ± 0.0	0.10	−0.02 [−0.1, 0.0]	0.68	0.70
eGFR ^$^ (mL/min/1.73 m^2^)	92.7 ± 3.2	94.3 ± 2.7	0.32	90.8 ± 3.2	95.1 ± 3.1	0.01	2.7 [−1.9, 6.5]	0.67	0.28

Data are expressed as mean ± SE ^$^ and median (interquartile range) ^γ^, mean difference [95% CI]), *p*-value; *n* = 60 (*n* = 30, 28 females and 2 males in each group). The intervention and the safety analysis were based on a modified intention-to-treat principle. The normally distributed data were analysed using ANCOVA, where within-group effects as intervention duration, between-group effects is the supplement type, and interaction effects (duration × supplement). The data which were not normally distributed were presented as median (IQR) and compared for within-group differences using Wilcoxon Signed rank test and for between-group differences using Mann–Whitney U test. At baseline, the physical, socioeconomic, and demographic characteristics were analysed by independent *t*-test, showing no significant differences (all *p* > 0.05). Abbreviations: CG, control group; SG, supplement group; CI, confidence intervals; FPG, fasting plasma glucose; HbA1c, glycated haemoglobin; HOMA-IR, homeostasis model assessment-estimated insulin resistance; LDL-C, low-density lipoprotein cholesterol; TC, total cholesterol; TG, triglycerides; HDL-C, high-density lipoprotein cholesterol; SGPT, serum glutamic-pyruvic transaminase; Cr, creatinine; eGFR, estimated glomerular filtration rate.

**Table 4:** Blood antioxidant, oxidant, and inflammatory markers before and after supplementation in both groups.

	CG			SG			Mean Difference [95% CI]	Between Groups at Baseline *p*-Value	Interaction Effects (Duration × Supplement) *p*-Value
	Before (*n* = 30)	After (*n* = 30)	Duration Effects (Within Group) *p*-Value	Before (*n* = 30)	After (*n* = 30)	Duration Effects (Within Group) *p*-Value			
Plasma vitamin C ^$^ (µmol/L)	38.8 ± 2.2	33.6 ± 2.6	0.07	38.2 ± 3.5	36.6 ± 3.7	0.56	3.6 [−4.0, 10.6]	0.88	0.37
Plasma MDA ^$^ (µmol/mL)	7.2 ± 0.8	6.3 ± 0.6	0.14	8.2 ± 0.9	7.1 ± 0.9	0.08	−0.2 [−1.3, 1.7]	0.46	0.82
Serum hsCRP ^γ^ (mg/L)	1.5 (1.0–4.3)	2.0 (1.0–5.3)	0.28	2.0 (1.0–3.0)	1.5 (1.0–3.0)	0.14	−0.3 [−0.8, 0.2]	0.90	0.07
WBC in plasma ^$^ (×10^9^/L)	9.8 ± 0.5	10.7 ± 0.6	0.12	10.4 ± 0.7	9.7 ± 0.4	0.38	−1.6 [−2.4, 0.2]	0.52	0.07
Neutrophil ^$^ (%)	55.7 ± 1.9	56.3 ± 1.5	0.74	54.9 ± 2.0	53.4 ± 1.6	0.39	−2.1 [−6.2, 1.2]	0.77	0.19
Lymphocyte ^$^ (%)	33.5 ± 1.7	33.0 ± 1.2	0.76	34.5 ± 1.4	36.0 ± 1.3	0.28	2 [−0.5, 5.7]	0.66	0.10
Monocyte ^$^ (%)	5.4 ± 0.2	5.4 ± 0.2	0.98	5.3 ± 0.2	5.4 ± 0.2	0.64	0.1 [−0.4, 0.5]	0.79	0.81
Eosinophil ^$^ (%)	5.2 ± 0.6	5.1 ± 0.7	0.93	5.1 ± 0.8	5.1 ± 0.8	0.94	0.1 [−0.7, 0.8]	0.91	0.92
Basophil ^$^ (%)	0.3 ± 0.1	0.2 ± 0.1	0.26	0.3 ± 0.1	0.2 ± 0.1	0.63	0.0 [−0.2, 0.2]	0.50	0.97

Data are expressed as mean ± SE ^$^, median (interquartile range) ^γ^, mean difference [95% CI], *p*-value; *n* = 60 (*n* = 30, 28 females and 2 males in each group). The intervention analysis was based on a modified intention-to-treat principle. The normally distribution data were analysed using ANCOVA, where within-group effects as intervention duration, between-group effects as the supplement type, and interaction effects (duration × supplement). The data that were not normally distributed were presented as median (IQR) and compared for within-group differences using Wilcoxon Signed rank test and for between-group differences using Mann–Whitney U test. At baseline, no significant differences between the groups were found for any of the variable (all *p* > 0.05). The group-wise comparisons are based on absolute values. Abbreviations: CG, control group; SG, supplement group; CI, confidence intervals; MDA, malondialdehyde; hsCRP, high sensitivity C-reactive protein; WBC, white blood cell.

**Table 5:** Variables of 6MWT of the participants before and after supplementation in both groups.

	CG			SG			Mean Difference [95% CI]	Between Groups at Baseline *p*-Value	Interaction Effects (Duration × Supplement) *p*-Value
	Before (*n* = 30)	After (*n* = 30)	Duration Effects (Within Group) *p*-Value	Before (*n* = 30)	After (*n* = 30)	Duration Effects (Within Group) *p*-Value			
6MWD ^$^ (m)	433.2 ± 10.2	432.2 ± 10.7	0.84	452.6 ± 11.6	443.7 ± 12.9	0.26	−7.9 [−25.1, 12.6]	0.21	0.51
VO_2peak_^$^ (mL/kg/min)	15.6 ± 0.4	15.6 ± 0.4	0.91	16.3 ± 0.5	16.0 ± 0.5	0.18	−0.3 [−0.7, 0.3]	0.27	0.34

Data are expressed as mean ± SE ^$^, mean difference [95% CI], *p*-value; *n* = 60 (*n* = 30, 28 females and 2 males in each group). The analysis was based on a modified intention-to-treat principle. The normally distribution data were analysed using ANCOVA, where within-group effects as intervention duration and between-group effects is the supplement type, and interaction effects (duration × supplement). At baseline, there was no significant differences between the two groups (all *p* > 0.05). The intervention did not significantly change any of the variables in either group, and there were no interaction effects (all *p* > 0.05). The group-wise comparisons are based on absolute values. Abbreviation: CG, control group; SG, supplement group; CI, confidence intervals; 6MWD, 6-minute walk distance; VO_2peak_, peak oxygen consumption.

## Data Availability

The original contributions presented in this study are included in the article/Supplementary Material. Further inquiries can be directed to the corresponding author.

## Supplementary Materials

The following supporting information can be downloaded at: https://www.mdpi.com/article/10.3390/nu17071098/s1, Supplement S1: Table S1: Consort checklist of this study; Table S2: A number of the participants took the medicines throughout the supplementations in both groups; Table S3: Amounts of active ingredients and antioxidant activity and their analytical methods of prebiotic supplement capsule; Table S4: Capsule consumption record form; Table S5: Thresholds of all variables in this study; Table S6: The number and percentage of capsule consumption throughout the supplementations in both groups; Figure S1: Glucose profile; Figure S2: Lipid profile. Supplement S2: Participants’ characteristics and health questionnaire form.

## Abbreviations

The following abbreviations are used in this manuscript:

T2DM	Type 2 Diabetes Mellitus
SG	Supplement group
CG	Control group
FPG	Fasting plasma glucose
HbA1c	Glycated haemoglobin A1c
LDL-C	Low-density lipoprotein cholesterol
TG	Triglycerides
HOMA-IR	Homeostasis model assessment-estimated insulin resistance
TC	Total cholesterol
HDL-C	High-density lipoprotein cholesterol
MDA	Malondialdehyde
hsCRP	High sensitivity C-reactive protein
WBC	White blood cell
6MWT	6-minute walk test
6MWD	6-minute walk distance
Cr	Creatinine
eGFR	Estimated glomerular filtration rate
SGPT	Serum glutamic-pyruvic transaminase
SBP	Systolic blood pressure
DBP	Diastolic blood pressure
MET	Metabolic equivalent of task
VO_2peak_	Peak oxygen uptake
