# Supplementary material for: Diabetes Therapeutics of Prebiotic Soluble Dietary Fibre and Antioxidant Anthocyanin Supplement in Patients with Type 2 Diabetes: Randomised Placebo-Controlled Clinical Trial
Source: Nutrients. 2025 Mar 21;17(7):1098. doi: 10.3390/nu17071098 (PMC11990404; doi:10.3390/nu17071098)
Supplement: Supplementary file 1 [file nutrients-17-01098-s001.zip › nutrients-3450777-supplementary.pdf]

Supplementary files

Supplement S1, Table S1. Consort checklist of this study.

Supplement S1, Table S2. A number of the participants took the medicines throughout the supplementations in both groups.

Supplement S1, Table S3. Amounts of active ingredients and antioxidant activity and their analytical methods of prebiotic supplement capsule.

Supplement S1, Table S4. Capsule consumption record form.

Supplement S1, Table S5. Thresholds of all variables in this study.

Supplement S1, Table S6. The number and percentage of capsule consumption throughout the supplementations in both groups.

Supplement S1, Figure S1. Glucose profile: FPG (mg/dL) (a) and plasma HbA1c (%) (b) concentrations before and after supplementation of the participants in both groups. The data are expressed as mean ± SE, p-value; n = 60 (n=30 each group). The interventions were based on a modified intention-to-treat principle and were analysed by ANCOVA. Abbreviations: CG, control group; SG, supplement group; FPG, fasting plasma glucose; HbA1c, glycated haemoglobin A1c.

Supplement S1, Figure S2. Lipid profile: Plasma LDL-C (a), TC (b), TG (c), and HDL-C (d) concentrations of the participants before and after supplementation in both groups. The data are expressed asmean ± SE, p value; n = 60 (n=30 each group). The interventions were based on a modified intention-to-treat principle and were analysed by ANCOVA. Abbreviations: CG, control group; SG, supplement group; LDL-C, low-density lipoprotein cholesterol; TC, total cholesterol; TG, triglycerides; HDL-C, high-density lipoprotein cholesterol.

Supplement S2. Participants' characteristics and health questionnaire form.

Supplement S1, Table S1. Consort checklist of this study

CONSORT 2010 checklist of information to include when reporting a randomised trial*

Section/Topic	Item No	Checklist item	Reported on page No
**Title and abstract**			
	1a	Identification as a randomised trial in the title	1
	1b	Structured summary of trial design, methods, results, and conclusions (for specific guidance see CONSORT for abstracts)	1-2
**Introduction**			
Background and objectives	2a	Scientific background and explanation of rationale	2-3
	2b	Specific objectives or hypotheses	3
**Methods**			
Trial design	3a	Description of trial design (such as parallel, factorial) including allocation ratio	3
	3b	Important changes to methods after trial commencement (such as eligibility criteria), with reasons	N/A
Participants	4a	Eligibility criteria for participants	4
	4b	Settings and locations where the data were collected	3
Interventions	5	The interventions for each group with sufficient details to allow replication, including how and when they were actually administered	5-8
Outcomes	6a	Completely defined pre-specified primary and secondary outcome measures, including how and when they were assessed	9-11

Section/Topic	Item No	Checklist item	Reported on page No
	6b	Any changes to trial outcomes after the trial commenced, with reasons	N/A
Sample size	7a	How sample size was determined	4
	7b	When applicable, explanation of any interim analyses and stopping guidelines	3
Randomisation:			
Sequence generation	8a	Method used to generate the random allocation sequence	5
	8b	Type of randomisation; details of any restriction (such as blocking and block size)	5
Allocation concealment mechanism	9	Mechanism used to implement the random allocation sequence (such as sequentially numbered containers), describing any steps taken to conceal the sequence until interventions were assigned	5
Implementation	10	Who generated the random allocation sequence, who enrolled participants, and who assigned participants to interventions	5
Blinding	11a	If done, who was blinded after assignment to interventions (for example, participants, care providers, those assessing outcomes) and how	5
	11b	If relevant, description of the similarity of interventions	5
Statistical methods	12a	Statistical methods used to compare groups for primary and secondary outcomes	11
	12b	Methods for additional analyses, such as subgroup analyses and adjusted analyses	11
**Results**			
Participant flow (a diagram is strongly recommended)	13a	For each group, the numbers of participants who were randomly assigned, received intended treatment, and were analysed for the primary outcome	3
	13b	For each group, losses and exclusions after randomisation, together with reasons	3

Section/Topic	Item No	Checklist item	Reported on page No
Recruitment	14a	Dates defining the periods of recruitment and follow-up	3
	14b	Why the trial ended or was stopped	Because of the completion of this research.
Baseline data	15	A table showing baseline demographic and clinical characteristics for each group	7,12,13,15,18,19
Numbers analysed	16	For each group, number of participants (denominator) included in each analysis and whether the analysis was by original assigned groups	3
Outcomes and estimation	17a	For each primary and secondary outcome, results for each group, and the estimated effect size and its precision (such as 95% confidence interval)	12,13,15,18,19
	17b	For binary outcomes, presentation of both absolute and relative effect sizes is recommended	N/A
Ancillary analyses	18	Results of any other analyses performed, including subgroup analyses and adjusted analyses, distinguishing pre-specified from exploratory	N/A
Harms	19	All important harms or unintended effects in each group (for specific guidance see CONSORT for harms)	17, Table 3. No harmful effect.
**Discussion**			
Limitations	20	Trial limitations, addressing sources of potential bias, imprecision, and, if relevant, multiplicity of analyses	21-22
Generalisability	21	Generalisability (external validity, applicability) of the trial findings	22
Interpretation	22	Interpretation consistent with results, balancing benefits and harms, and considering other relevant evidence	20-22
**Other information**			

Section/Topic	Item No	Checklist item	Reported on page No
Registration	23	Registration number and name of trial registry	3
Protocol	24	Where the full trial protocol can be accessed, if available	3
Funding	25	Sources of funding and other support (such as supply of drugs), role of funders	23

Citation: Schulz KF, Altman DG, Moher D, for the CONSORT Group. CONSORT 2010 Statement: updated guidelines for reporting parallel group randomised trials. BMC Medicine. 2010;8:18. © 2010 Schulz et al. This is an Open Access article distributed under the terms of the Creative Commons Attribution License (http://creativecommons.org/licenses/by/2.0), which permits unrestricted use, distribution, and reproduction in any medium, provided the original work is properly cited.

*We strongly recommend reading this statement in conjunction with the CONSORT 2010 Explanation and Elaboration for important clarifications on all the items. If relevant, we also recommend reading CONSORT extensions for cluster randomised trials, non-inferiority and equivalence trials, non-pharmacological treatments, herbal interventions, and pragmatic trials. Additional extensions are forthcoming: for those and for up-to-date references relevant to this checklist, see www.consort-statement.org.

Supplement S1, Table S2. A number of the participants took medicines throughout the supplementations in both groups.

	**CG**	**SG**
**Hypoglycemic drug**		
Metformin hydrochloride (500 mg)	29	28
Glibenclamide (5 mg)	1	-
Glipizide (5 mg)	10	12
Insulin suspension for injections	2	-
Pioglitazone hydrochloride (15 mg)	2	-
Sitagliptin phosphate (50 mg)/Metformin hydrochloride (1000 mg)	1	-
**Antihypertensive drug**		
Enalapril maleate (5 mg)	5	4
Manidipine hydrochloride (20 mg)	1	1
Losartan potassium (50 mg)	-	1
Atenolol (50 mg)	-	1
Amlodipine (5 mg)	3	2
Hydrochlorothiazide (250 mg)	1	-
Aspirin (81 mg)	3	1
**Antidyslipidemic drugs**		
Simvastatin (20 mg)	8	6
Fenofibrate (300 mg)	1	-

Supplement S1, Table S3. Amounts of active ingredients and antioxidant activity and their analytical methods of prebiotic supplement capsule.

	mg/100 g	g/kg/d	Analytical methods
Soluble dietary fibre (rice bran)	4,238.37	0.872 ± 0.159	The analysis of soluble dietary fibre from dried RD6 rice bran powder was performed using Megazyme test kit (Megazyme International Ireland Ltd., Wicklow, Ireland) following the key components of the Association of Official Analytical Chemists (AOAC, 2012) [22] method (in the key components of 985.29. Soluble dietary fibre in rice bran was expressed as mg of inulin per 100 g sample (d.b.) [23].
Soluble dietary fibre (inulin)	19.30	0.004 ± 0.001	The analysis of inulin from the dried Jerusalem artichoke powder was performed using the Association of Official Analytical Chemists (AOAC, 2012) [22] method (in the key components of 999.03) using Megazyme kit (Megazyme International Ireland Ltd., Wicklow, Ireland). Soluble dietary fibre in inulin was expressed as mg of inulin per 100 g sample (d.b.) [23].
Anthocyanins	33.56	0.015 ± 0.003	The analysis of anthocyanin from dried extract anthocyanins powder was performed using the pH-differential method described by Lee and colleagues (2005) [24]. A 0.5 mL aliquot of the extract was diluted with 3.8 mL of buffer reagents (0.025 M potassium chloride (KCl) at pH 1.0 and 0.4 M sodium acetate ($C_2H_3NaO_2$) at pH 4.5), incubated in the dark condition for 20 min, then measured anthocyanin content using spectrophotometer (Shimadzu UV-Vis spectrophotometer UV-2600 with TCC-controller, Columbia, MD, USA) at absorbance 520 and 700 nm, respectively. Anthocyanins was expressed as mg cyanidin-3-

	mg/100 g	g/kg/d	Analytical methods
			glucoside equivalent (molecular weight of 449.2 g/mol and molar extinction coefficient of 29,600 M^{-1} cm^{-1}) per 100 g sample (mg Cy3G/100 g, d.b.) [25].
Antioxidant activity	904.35	0.041 ± 0.008	The analysis of anthocyanin from dried extract anthocyanins powder was performed using by (2,2-azinobis (3-ethylbenzothiazoline-6-sulfonic acid) (ABTS) Assay) described by Stratil and colleagues (2006) [26] with some modifications. In the dark condition at room temperature, the stock solution was prepared by reacting 7 mM of ABTS with 4.95 mM of potassium persulphate ($K_2O_8S_2$) with the ratio 1:1 (v/v) for 12 h. Working solution was generated by reacting the stock solution with phosphate buffer saline (PBS, pH 7.4) to absorbance 1.0 AU at 734 nm using spectrophotometer (Shimadzu UV-Vis spectrophotometer UV-2600 with TCC-controller, Columbia, MD, USA). Before analysis, a 40 µL of sample (mixed extract powders in prebiotic capsule) was reacted with 4 mL of the working solution and then incubated for 10 min in the dark. Antioxidant activity was expressed as mg Trolox equivalent per 100 g sample based on dry basis (mg TE/100 g, d.b.) [24].

Reference

29. Association of Official Analytical Chemists. *Official Method of Analysis*, 19th ed.; Association of Official Analytical Chemists: Washington, DC, USA, 2012.

30. Puyanda, I.R.; Uriyapongson, S.; Uriyapongson, J. Influence of drying method on qualities of Jerusalem artichoke (*Helianthus tuberosus* L.) tuber harvested in Northeastern Thailand. *Songklanakarin J. Sci. Technol.* **2020,** 42, 1279-1285.

31. Kapcum, C.; Uriyapongson, S.; Uriyapongson, J. Phenolics, anthocyanins and antioxidant activities in waste products from different parts of purple waxy corn (*Zea mays* L.). *Songklanakarin J. Sci. Technol*. **2021,** 43, 398-405.

32. Lee, J.; Durst, R.W.; Wrolstad, R.E. Determination of total monomeric anthocyanin pigment content of fruit juices, beverages, natural colorants, and wines by the pH differential method: Collaborative study. *J. AOAC Int.* **2005,** 88, 1269-1278.

33. Stratil, P.; Klejdus, B.; Kuban, V. Determination of total content of phenolic compounds and their antioxidant activity in vegetables evaluation of spectrophotometric methods. *J. Agric. Food Chem.* **2006**, 54, 607-616.

ID Code…………………………………….

Supplement S1, Table S4. Capsule consumption record form.

You will take 2 capsules/time, 4 times/day after breakfast, lunch, dinner, and before bedtime (total capsules are 8 capsules/day).

Fill ✓ if you take the capsules, fill X if you do not, and provide the reason. (If you forget to take any capsules, please keep the remaining capsules and return them to the researcher on the 30th).

**Day**	**Date**	**breakfast**	**lunch**	**dinner**	**bedtime**	**Note**	**Day**	**Date**	**breakfast**	**lunch**	**dinner**	**bedtime**	**Note**
1							16						
2							17						
3							18						
4							19						
5							20						
6							21						
7							22						
8							23						
9							24						
10							25						
11							26						
12							27						
13							28						
14							29						
15							30						

ID Code…………………………………….

Supplement S1, Table S4 (Continued). Capsule consumption record form.

You will take 2 capsules/time, 4 times/day that are immediately after breakfast, lunch, dinner, and before bedtime (total capsules are 8 capsules/day).

Fill ✓ if you take the capsules, fill X if you do not, and provide the reason. (If you forget to take any capsules, please keep the remaining capsules and return them to the researcher on the 60th).

Day	Date	breakfast	lunch	dinner	bedtime	Note	Day	Date	breakfast	lunch	dinner	bedtime	Note
31							46						
32							47						
33							48						
34							49						
35							50						
36							51						
37							52						
38							53						
39							54						
40							55						
41							56						
42							57						
43							58						
44							59						
45							60						

Supplement S1, Table S5. Thresholds of all variables in this study.

Blood variable	Threshold	Reference
Fasting blood glucose (mg/dL)	1.00	User's Manual for YSI 2300 STAT Plus™ Glucose and Lactate Analyser
Plasma HbA1c (%)	4.00	cobas HbA1c Test
Serum insulin (μIU/mL)	0.60	MP Biomedical; Endocrinology and Neuroscience
Serum hsCRP (mg/L)	3.00	cobas CRP Test
Plasma TC (mg/dL)	100.00	Reflotron Reagents
Plasma TG (mg/dL)	70.00	Reflotron Reagents
Plasma HDL-C (mg/dL)	10.00	Reflotron Reagents
Serum SGPT (U/L)	2.66	Reflotron Reagents
Plasma Cr (mg/dL)	0.50	Reflotron Reagents
WBC in plasma (x10^3/μL)	0.00	Routine Use Training Workbook XS-1000i
Plasma vitamin C (μmol/mL)	0.08	Zhang et al., 2009
Plasma MDA (μmol/mL)	0.50	Draper et al., 1993

Supplement S1, Table S6. The number and percentage of capsule consumption throughout the supplementations in both groups.

Participant	Group	Consumed capsules	Consumed capsules	Remaining capsules	Remaining capsules
		Number	Percentage	Number	Percentage
1	CG	474	98.8	6	1.3
2	CG	464	96.7	16	3.3
3	CG	460	95.8	20	4.2
4	CG	464	96.7	16	3.3
5	CG	462	96.3	18	3.8
6	CG	472	98.3	8	1.7
7	CG	468	97.5	12	2.5
8	CG	466	97.1	14	2.9
9	CG	462	96.3	18	3.8
10	CG	460	95.8	20	4.2
11	CG	466	97.1	14	2.9
12	CG	456	95.0	24	5.0
13	CG	462	96.3	18	3.8
14	CG	468	97.5	12	2.5
15	CG	464	96.7	16	3.3
16	CG	458	95.4	22	4.6
17	CG	462	96.3	18	3.8
18	CG	452	94.2	28	5.8
19	CG	466	97.1	14	2.9
20	CG	464	96.7	16	3.3
21	CG	460	95.8	20	4.2
22	CG	468	97.5	12	2.5
23	CG	464	96.7	16	3.3
24	CG	472	98.3	8	1.7
25	CG	468	97.5	12	2.5

26	CG	466	97.1	14	2.9
27	CG	472	98.3	8	1.7
28	CG	470	97.9	10	2.1
29	CG	462	96.3	18	3.8
30	CG	464	96.7	16	3.3
	**Total**	**464.5**	**96.8**	**15.5**	**3.2**

Abbreviation: CG, control group

Supplement S1, Table S6 (Continued). The number and percentage of capsule consumption throughout the supplementations in both groups.

Participant	Group	Consumed capsules	Consumed capsules	Remaining capsules	Remaining capsules
		Number	Percentage	Number	Percentage
1	SG	464	96.7	16	3.3
2	SG	468	97.5	12	2.5
3	SG	466	97.1	14	2.9
4	SG	470	97.9	10	2.1
5	SG	458	95.4	22	4.6
6	SG	472	98.3	8	1.7
7	SG	468	97.5	12	2.5
8	SG	460	95.8	20	4.2
9	SG	472	98.3	8	1.7
10	SG	474	98.8	6	1.3
11	SG	470	97.9	10	2.1
12	SG	468	97.5	12	2.5
13	SG	478	99.6	2	0.4
14	SG	470	97.9	10	2.1
15	SG	466	97.1	14	2.9
16	SG	478	99.6	2	0.4
17	SG	472	98.3	8	1.7
18	SG	468	97.5	12	2.5
19	SG	470	97.9	10	2.1
20	SG	472	98.3	8	1.7
21	SG	474	98.8	6	1.3
22	SG	466	97.1	14	2.9
23	SG	470	97.9	10	2.1
24	SG	472	98.3	8	1.7
25	SG	478	99.6	2	0.4

26	SG	464	96.7	16	3.3
27	SG	468	97.5	12	2.5
28	SG	474	98.8	6	1.3
29	SG	470	97.9	10	2.1
30	SG	468	97.5	12	2.5
	**Total**	**469.6**	**97.8**	**10.4**	**2.2**

Abbreviation: SG, supplement group

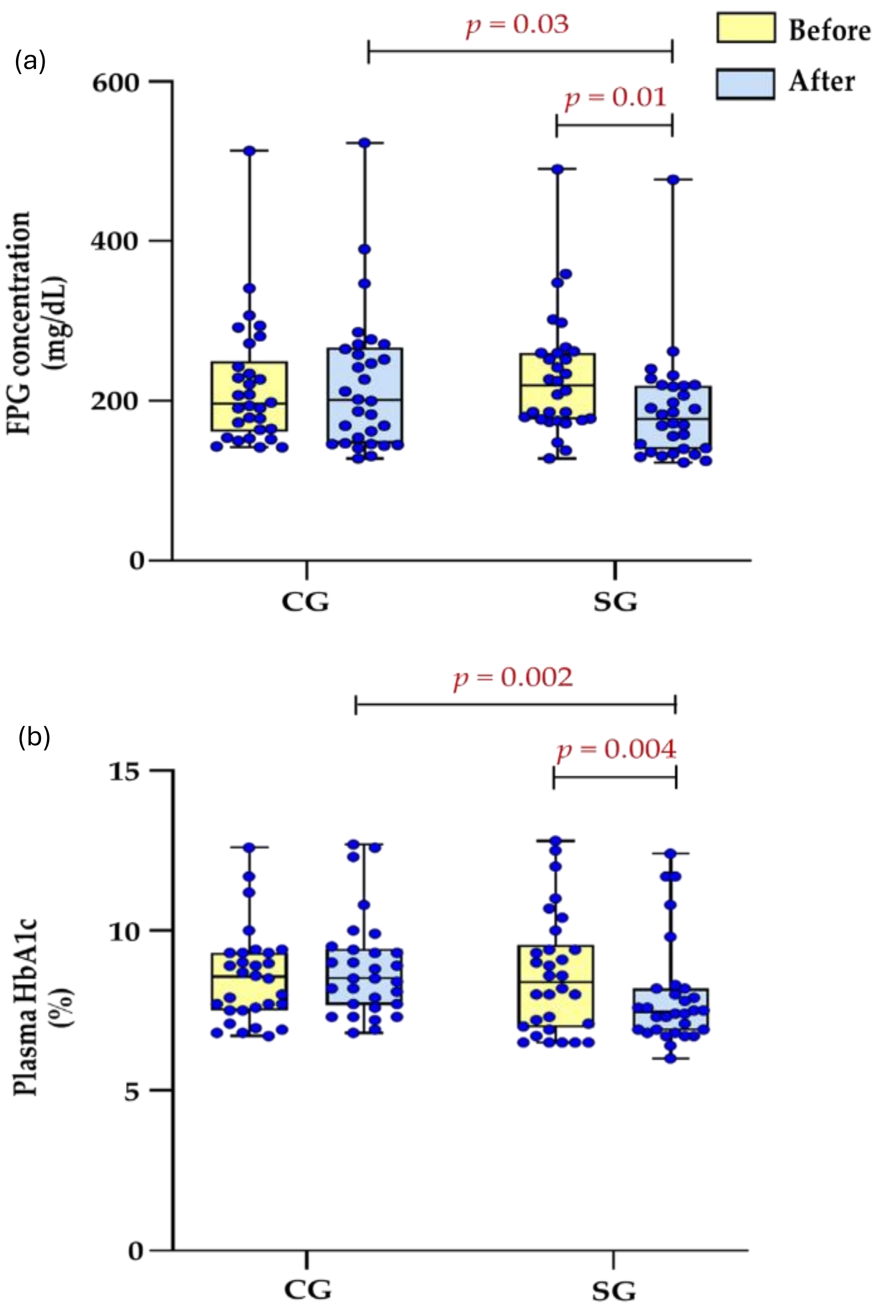

Figure S1. Glucose profile: FPG (mg/dL) (a) and plasma HbA1c (%) (b) concentrations before and after supplementation of the patients in both groups. The data are expressed as the mean ± SE; n = 60 (n=30 each group). The interventions were based on a modified intention-to-treat principle and were analysed using ANCOVA. Abbreviations: CG, control group; SG, supplement group; FPG, fasting plasma glucose; HbA1c, glycated haemoglobin A1c.

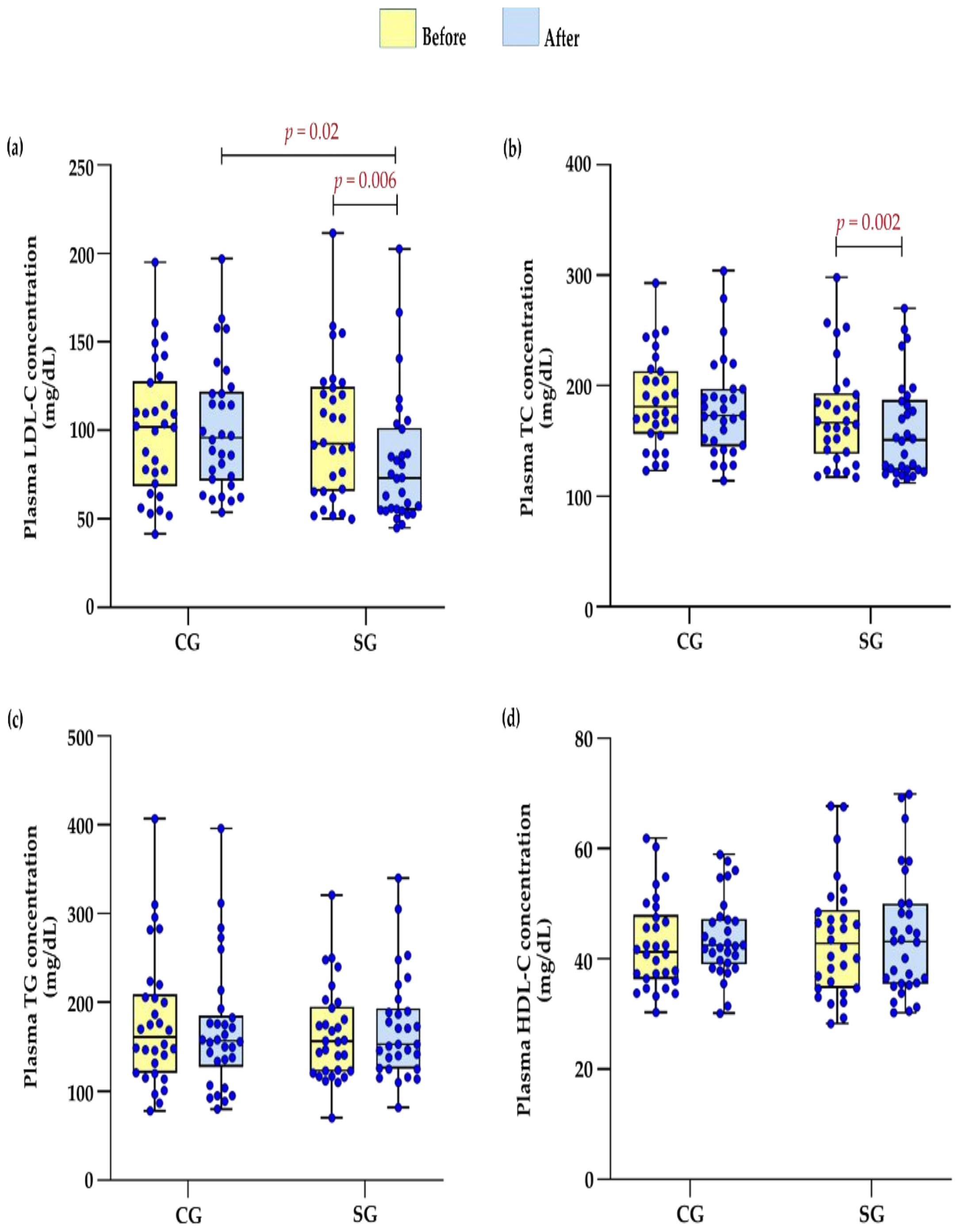

Supplement S1, Figure S2. Lipid Profile: Plasma LDL-C (a), TC (b), TG (c), and HDL-C (d) concentrations of the patients before and after supplementation in both groups. The data are expressed as the mean ± SE, p value); n = 60 (n=30 each group). The interventions were based on a modified intention-to-treat principle and were analysed using ANCOVA. Abbreviations: CG, control group; SG, supplement group; LDL-C, low-density lipoprotein cholesterol; TC, total cholesterol; TG, triglycerides; HDL-C, high-density lipoprotein cholesterol.

Supplement S2. Participant's Characteristics and Health Questionnaire Form

"Effects of dietary supplement containing anthocyanins, inulin, and rice bran dietary fiber on oxidative stress, inflammation, insulin resistance, fat oxidation and cardiovascular function in patients with type 2 diabetes mellitus"

Please fill in your information. They will be kept secret and used only for this research.

ID Code…………………………………….

Date...........Month..........................Year............

1. Name-surname...
2. Sex ( ) male ( ) female
3. Career ....
4. Birth date……..... Month…………..……. Year................................
5. Age ....Year .... Month ....
6. Address...
7. Body weight .... kg. Body height ....cm.
8. Marital status ( ) Single ( ) Married ( ) divorce

   Number of children...................
9. Underlying diseases

   ( ) Cardiovascular disease Please specify…………

   ( ) Neurological disease Please specify…………

   ( ) Diabetes type 2

   ( ) Musculoskeletal disease Please specify…………

   ( ) Respiratory disease Please specify …………

   ( ) Surgery.......................................Date....................

   ( ) Others...

   ( ) No
10. Medication (name, number, and frequency/day) ....
11. Underlying diseases of your family

    ( ) No ( ) Yes Please specify ……………………………………………
12. Have you had an allergy to the following?

    a) Rice (white and riceberry)

b) Jerusalem artichoke

c) Others, please specify …………………………………..

13. Smoking ( ) Don't smoke ( ) Smoke

Please specify the number and frequency/day for how long ....

14. Drink ( ) Don’t drink ( ) Drink

Please specify the quantity and frequency/day for which it will last ....

15. Drug addict ( ) Don’t take ( ) Take

Please specify the quantity and frequency/day for which it will last ....

16. History of exercise

16.1. In the past 4 months, how often did you regularly exercise per week............................

16.2. What type of exercise? ……………………………….

Please define whether you are a sports player ……………………………………………

16.3. What intensity of the exercise

( ) low (can speak and sing) ( ) moderate (cannot speak but can sing) ( ) high (cannot talk and cannot sing)

16.4. Have you ever had an injury from the exercise

( ) No ( ) Yes Please specify ..……………………….

17. History of nutritional supplements in the past 1 year

( ) No ( ) Yes

Please specify ………………………………………

Now, you are taking the supplement

( ) No ( ) Yes

Please specify its/their name(s) and number(s) and frequency …..……………………

Signature...

(..)

Date..........Month........................Year................

We thank you for your kind report.
